# Safety and efficacy of addition of hyaluronidase to a mixture of lidocaine and bupivacaine in scalp nerves block in elective craniotomy operations; comparative study

**DOI:** 10.1186/s12871-018-0590-9

**Published:** 2018-09-15

**Authors:** Ahmed Abdalla Mohamed, Tarek Ahmed Radwan, Mohamed Mahmoud Mohamed, Hatem Abdellatif Mohamed, Mohamed Farid Mohamed Elemady, Safinaz Hassan Osman, Tamer Fayez Safan, Tamer Mohamed Khair, Norhan Abdelaleem Ali, Rania Samir Fahmy, Mohamed Ibrahim Belita, Shady Rady Abdalla, Ahmed Abdelaziz Seleem, Ehab Mohi Atta, Osama Ismail Zaid, Ahmed Shaker Ragab, Ahmed Essam Salem, Badawy Mohamed AlKholy

**Affiliations:** 1 Anaesthesia, Faculty of Medicine, Cairo University, 139 H,Kafra Gate,Hadayk AL Ahram, Giza, Egypt; 2 Anaesthesia, Faculty of Medicine, Cairo University, 52 Elthawra street, Heliopolis, Cairo, Egypt; 3 Anaesthesia, Faculty of Medicine, Cairo University, 10 Abdlhameed Kotb Street, Kozzika, Maadi, Cairo, Egypt; 4 Anaesthesia, Faculty of Medicine, Cairo University, 6 Ahmed Angad Street, Faisal-Alharam-Giza, Egypt; 5 Anaesthesia, Faculty of Medicine, Cairo University, 391 Sudan Street, El Mohandsen, Cairo, Egypt; 6 Anaesthesia, Faculty of Medicine, Cairo University, 4 Hussein El Ezaby Street, Misr Alexandria Road, Cairo, Egypt; 7 Anaesthesia, Faculty of Medicine, Cairo University, 1 Alsaraya Street, Almanyal, 11559, Cairo, Egypt; 8 Anaesthesia, Faculty of Medicine, Cairo University, 290 Teraat el gabal Street, El Zayton, Cairo, Egypt; 9 Anaesthesia, Faculty of Medicine, Cairo University, 29 Mansoura street, Agouza, Giza, Egypt; 10 Anaesthesia, Faculty of Medicine, Cairo University, 1 Alsaraya Street, Almanyal, 11559, Cairo, Egypt; 11 Anaesthesia, Faculty of Medicine, Cairo University, 15 B, Misr Construction Buldings,Zahraa el Maadi, Cairo, Egypt; 12 Anaesthesia, National Cancer Institute, Anaesthesiology Department, Cairo University, Kasr Alainy Street, Cairo, Egypt; 13 Anaesthesia, Faculty of Medicine, Tanta University, 92 Ahmed Orabi Street, El Mohandsen, Cairo, Egypt; 14 Chemical Patholpgy, Faculty of Medicine, Cairo University, 10 B Youssef Algendy Street, Bab Allouk, Cairo, Egypt

**Keywords:** Scalp block, Craniotomy, Bupivacaine, Lidocaine, Hyaluronidase

## Abstract

**Background:**

Patients undergoing craniotomy operations are prone to various noxious stimuli, many strategies are commenced to provide state of analgesia, for better control of the stress response and to overcome its undesired effects on the haemodynamics and post-operative pain. Scalp nerves block are considered one of these strategies. This study was conceived to evaluate the effect of addition of hyaluronidase to the local anaesthetic mixture used in the scalp nerves block in patients undergoing elective craniotomy operations.

**Methods:**

64 patients undergoing elective craniotomy operations were enrolled in this prospective randomized, double-blind comparative study. Patients were randomly assigned to two groups. Group LA, patients subjected to scalp nerves block with 15 ml bupivacaine 0.5%, 15 ml lidocaine 2%, in 1:400000 epinephrine. Group H as Group LA with15 IU /ml Hyaluronidase.

**Results:**

Patients in the H group showed lower VAS values for 8 h postoperative, compared to the LA group. The haemodynamic response showed lower values in the H group, compared to the LA group. Those effects were shown in the intraoperative period and for 6 h post-operative. No difference was detected regarding the incidence of complications nor the safety profile.

**Conclusion:**

Our data supports the idea that addition of hyaluronidase to the local anesthetic mixture improves the success rates of the scalp nerves block and its efficacy especially during stressful intraoperative periods and in the early postoperative period. No evident undesirable effects in relation to the addition of hyaluronidase.

**Trial registration:**

Clinical Trial registry on ClinicalTrials.gov, NCT 03411330, 25-1-2018.

## Background

Patients undergoing craniotomy operations are susceptible to many injurious and painful stimuli such as skin incision, insertion of cranial pins, Dural incision, Dural and skin closure. The use of corticosteroids, site and type of operation as well as the age and sex may interfere with pain severity. Strategies to blunt and attenuate the stress response to these noxious stimuli include administration of systemic opioids, deepening the level of anaesthesia and scalp nerve blocks [1, 2].

Using regional anaesthetic techniques in addition to general anaesthesia have been conducted to benefit from multimodality managements for post-craniotomy pain and to decrease systemic administration of analgesics, hence to decrease their systemic undesired implications [2].

The stress response is the hormonal and metabolic changes that follow injury or trauma. This includes wide range of endocrinal and immunological effects. The stress response to surgery is characterized by increased secretion of pituitary hormones and activation of the sympathetic nervous system. Consequently, catecholamines secretion from adrenal medulla increase along with norepinephrine release from presynaptic nerve terminals [3, 4]. Blocking the scalp nerves using 0.5% bupivacaine combined with lidocaine 2%is readily used to decrease hemodynamic response during and after craniotomy operations [5, 6].

In the current study, the effects of adding hyaluronidase to the local anesthetic mixture in scalp nerves block were observed, in patients undergoing relatively non-lengthy craniotomy operations.

## Methods

**Fig. 1:**
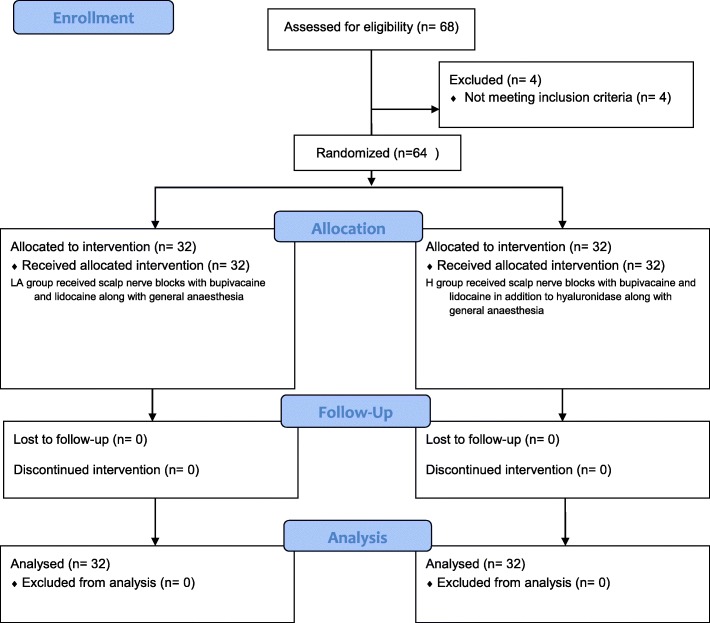
Consort Flow diagram

After approval by local department and hospital scientific and institutional ethics committee (number N-58/2017), this prospective, randomized, double-blind, comparative trial was conducted from February 2018 to April 2018. This prospective randomized clinical trial was registered in ClinicalTrials.gov (ID: NCT03411330), afterwards it was conducted in neurosurgery theatre of the Cairo university hospitals after obtaining written informed consent from all participants before enrolment in the study. The study was carried out on 64 American Society of Anesthesiologists (ASA) I-II patients aged between 18 and 60 years, undergoing elective supratentorial craniotomy operations under GA. Operations were performed in the supine position with estimated time range 4–6 h. Patients undergoing surgeries to remove pituitary tumors or lasting more than 6 h were excluded. Patients refusing operation, with history of allergy to the used drugs or postoperative GCS less than 12 on arousal with no improvement till admission to ICU were also excluded as investigators were not able to assess VAS. (Fig. 1).

All patients were told about the procedure, advantages and risks during the preoperative assessment done 1 day prior to surgery. Upon the arrival to the operating theater five lead ECG, pulse oximetry, non-invasive blood pressure and invasive blood pressure were applied to all patients. The latter measured by a transducer connected to arterial catheter which was inserted preoperatively in the non-dominant hand under local anesthesia after negative modified Allen’s test,. Peripheral nerve stimulator was added to monitor the degree of muscle relaxation.

Anaesthesia was induced by Propofol (2 mg/kg), (fentanyl 2 μg/kg) and intubation facilitated by using atracurium (0.5 mg/kg). Central line was inserted in the right internal jugular vein.

### Scalp nerves block technique

The patients were randomized into two groups using computer generated random number table. All patients in LA group (local anesthetic group) and H group (hyaluronidase added to local anesthetic group) received scalp nerves block using 15 ml of bupivacaine 0.5% with 15 ml lidocaine 2%, epinephrine added to this mixture (2.5 μg/mL) concentration i.e. 1:400000. In H group, hyaluronidase in a dose of 15 IU/ml was added to the local anesthetic mixture. Sealed envelopes were opened by an anesthesiologist not involved in the study to prepare the drug solution according to randomization. The observer who collected the peri-operative data as well as the patients were blinded to the drugs administered.

The following nerves were blocked taking in consideration that the whole volume did not exceed 30 ml, each nerve was blocked with 2 to 3 ml of the prepared mixture:

- Supraorbital nerve, the supraorbital foramen was palpated by the finger, and the needle is inserted along the upper orbital margin, perpendicular to the skin, approximately 1 cm medial to the supraorbital foramen.
- Supratrochlear nerve, blocked as it emerges above the eyebrow.
- Zygomaticotemporal nerve, blocked by infiltration from the supraorbital margin to the posterior part of the zygomatic arch.
- Auriculotemporal nerve, blocked by infiltration over zygomatic process, with an injection 1 to1.5 cm anterior to the ear at the level of the tragus.
- Greater occipital nerve, blocked by infiltration approximately halfway between the occipital protuberance and the mastoid process, 2.5 cm lateral to the nuchal median line.
- Lesser occipital nerve, blocked by infiltration along the superior nuchal line, 2.5 cm lateral to the greater occipital nerve block.
- Greater auricular nerve, blocked separately or with the injection of the occipital nerves. Inject the local anaesthetic about 2 cm posterior to the auricle, at the level of the tragus.

Bilateral block was done, with supplemental infiltration of the skin incision as well as the temporalis fascia. Lidocaine only soaked gauzes were used to block the dura.

### Maintenance

Anaesthesia maintained with isoflurane (1.2% end tidal) and 100% O2 changed to O2 air (1:1) after induction. Ventilation adjusted to maintain an end tidal CO2 between 30 and 35 mmHg. Muscle relaxation maintained by an infusion of atracurium (5–10 μg /kg/min). All patients receive intraoperative fluid in the form of normal saline infusion (3 ml/kg/hr.) and voluven (10 ml/kg). Dexamethasone 8 mg was given as a bolus to all patients. Blood loss was replaced if necessary with equal volumes of PRBCs and FFP. For patients with elevated blood pressure and heart rate 20% of baseline, additional dose of fentanyl (1 μg/kg) was given. The number and percent of these cases were recorded.

### Emergence and recovery

Atracurium infusion was discontinued after closure of the dura and isoflurane was discontinued after skin closure. Neuromuscular blockade was reversed with neostigmine (0.05 mg/kg) and atropine 0.01 mg/kg. Extubation was done at TOF ratio 0.9. All patients were discharged to the ICU for post-operative monitoring.

### Post-operative

Any patient with VAS score (VAS > or = 5) indicating moderate to severe pain received rescue analgesia in the form of 30 mg ketorolac IV infusion with maximum 120 mg in 24 h; time of the first analgesic request was recorded.

#### Measurement tools

Heart rate (HR), systolic (SBP), diastolic (DBP), mean (MAP) arterial blood pressures were recorded at the following times: 5 min before the block, 5 min after the block, 5 min after skin incision, every 30 min for 2 h intraoperative, 5 min after dural closure, 5 min after skin closure, every 30 min for 6 h postoperative then every 2 h for 24 h.Number of patients requiring intraoperative rescue doses of opioids were recorded. Pain was assessed using VAS postoperatively every 2 h for 24 h as well as obtaining the time for first analgesic requirement. Interleukin 6 basal level was measured before surgery (9:00 A.M), 30 min after scalp nerves block, 60 min after skin incision and after 6 h post-operative. Serum samples were collected, centrifuged within 1 h, frozen at − 20 °C or lower,

### Study outcomes

#### Primary outcome

Post-operative VAS for pain every 2 h for 24 h.

#### Secondary outcomes

1. Effects on hemodynamics: Heart rate, systolic, diastolic and mean arterial blood pressure intra and post-operative in both groups. 5 min before the block, 5 min after the block, 5 min after skin incision, every 30 min for 2 h intraoperative, 5 min after Dural closure, 5 min after skin closure, every 30 min for 6 h postoperative then every 2 h for 24 h.
2. Measurement of the level of interleukin 6 as an indicator of inflammatory response and pain. Basal level was measured before surgery, 30 min after scalp nerves block, 60 min after skin incision and after6 hours post-operative.
3. Number and percent of cases requiring intraoperative rescue dose of opioids.
4. Time of first analgesic request, Time of breakthrough pain felt by the patient that necessitated rescue analgesia.
5. Haemoglobin level after craniotomy before bone flap was done (to assess blood loss due to the VD effect of hyaluronidase).

### Statistical methods

Data were coded and entered using the statistical package SPSS version 25. Data was summarized using mean and standard deviation for normally distributed quantitative variables or median and interquartile range for non-normally distributed quantitative variables and frequencies (number of cases) and relative frequencies (percentages) for categorical variables [7]. Comparisons between groups were done using analysis of variance (ANOVA) with multiple comparisons post hoc test in normally distributed quantitative variables while non-parametric Kruskal-Wallis test and Mann-Whitney test were used for non-normally distributed quantitative variables (Chan, 2003a). For comparing categorical data, Chi square ( [2] test was performed [8]. Exact test was used instead when the expected frequency is less than 5 (Chan, 2003b). For comparison of serial measurements within each group repeated measures ANOVA was used in normally distributed quantitative variables while non-parametric Friedman test was used for non-normally distributed quantitative variables (Chan, 2004). *P*-value less than 0.05 was considered as statistically significant [9].

### Sample size

Total sample size of 64 patients randomly allocated into two equal groups. According to study “Hyaluronidase in sub-Tenon’s anesthesia for phacoemulsification” that was done and published in International Journal of Ophthalmology (2012) to detect difference in post-operative pain between those patients receiving Lidocaine only versus Lidocaine and hyaluronidase [10] where mean VAS was significantly higher in the group receiving LA only than in the group receiving LA and hyaluronidase; indicating more severe pain in the former (mean difference was 3.00 ± 1.55 and1.90 ± 1.45in the control and hyaluronidase groups respectively), sample size of 32 patients per group is needed with confidence level 95% power of the study 80% & type I error 0.05. Sample size calculation was done using Medcalc Software version 15.4.

## Results

Sixty-eight patients, scheduled for elective craniotomy agreed on participating in the study; of these four patients were excluded for not meeting the inclusion criteria. Thereby, remaining 64 were randomly divided into two groups. All patients completed the study.

### Demographic data

There were no significant differences in demographic and operative data between the 2 groups about patients’ age, gender, type and side of the operation (Table 1).

**Table 1:** Demographic data, ASA and operative data

Age (Years)	LA group 50.06 ± 11.17		H group 49.13 ± 10.33		*P* value 0.729
Gender					
Male	18	56.2%	17	53.1%	0.802
Female	14	43.8%	15	46.9%	
ASA					
I	21	65.6%	23	71.9%	0.590
II	11	34.4%	9	28.1%	
Side of operation					
Right	16	50.0%	21	65.6%	0.206
Left	16	50.0%	11	34.4%	
Type of the opreration					
Frontal glioma	14	43.75%	10	31.25%	
Parasagital meningioma	5	15.62%	4	12.5%	
Temporal glioma	9	28.12%	11	34.37%	0.8046
Aneurysm clipping	1	3.1%	2	6.25%	
Temporal meningiomas	1	3.1%	3	9.37%	
Frontal meningiomas	2	6.25%	2	6.25%	

Values are represented as as mean ± SD or count and percent as appropriate

*(H)* Meaning for the hyaluronidase group

*(LA)* Meaning for the local anesthetics only group

### The VAS for pain

GCS in all patients upon arrival to the ICU was 14–15 consequently, none of the patients was excluded from VAS assessment. There were significant statistical differences between the 2 groups, where the VAS for pain was lower in the hyaluronidase group immediately post-operative, after 2, 4, 6 and 8 h post operatively. However, there were no significant statistical differences in the VAS for pain afterwards and until the end of the 24 h as showed in Table 2.

**Table 2:** VAS for pain, comparison between the two groups

VAS/Hour	LA group	H group	*P* value
VAS 0	5.19 ± 1.4	3.22 ± 1.24	< 0.001*
VAS 2	4.88 ± 1.07	3.31 ± 1.09	< 0.001*
VAS 4	4.88 ± 1.01	3.5 ± 0.62	< 0.001*
VAS 6	5.31 ± 0.9	3.75 ± 0.8	< 0.001*
VAS 8	6.34 ± 1.07	4.72 ± 0.89	< 0.001*
VAS 10	4.41 ± 1.04	4 ± 0.8	0.12
VAS 12	6.09 ± 0.89	6.44 ± 1.08	0.111
VAS 14	5.06 ± 0.8	4.72 ± 1.67	0.323
VAS 16	6 ± 0.8	5.81 ± 0.78	0.39
VAS 18	5.72 ± 0.96	5.81 ± 0.9	0.693
VAS 20	5.19 ± 0.82	5.59 ± 0.87	0.06
VAS 22	5.75 ± 0.88	5.75 ± 0.98	0.955
VAS 24	5.62 ± 0.94	5.53 ± 0.84	0.605

*(H)* Meaning for the hyaluronidase group

*(LA)* Meaning for the local anesthetics only group

Values are represented as mean ± SD

*Significant *P* value less than (0.05), comparison between the 2 groups

### As regard the hemodynamic parameters

**Fig. 2:**
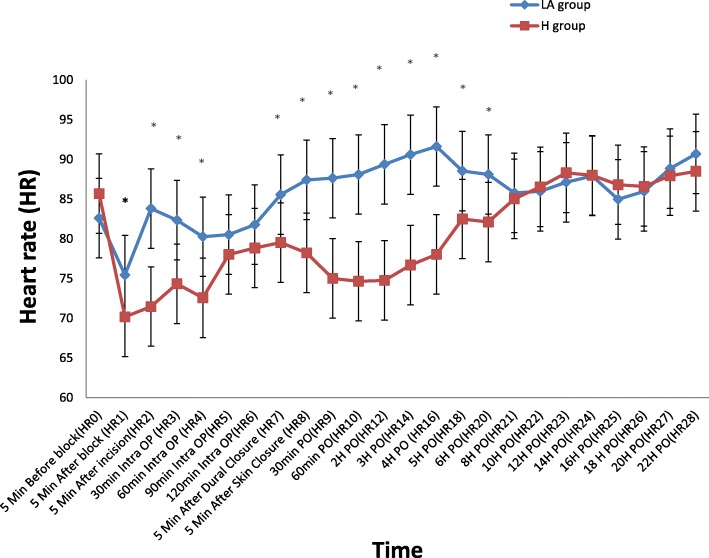
Comparison between the 2 groups as regards perioperative and intraoperative heart rate (HR). *(H) Meaning for the hyaluronidase group, (LA) Meaning for the local anesthetics only group, Values are represented as mean ± SD,* * Significant *P* value less than (0.05), comparison between the 2 groups

**Fig. 3:**
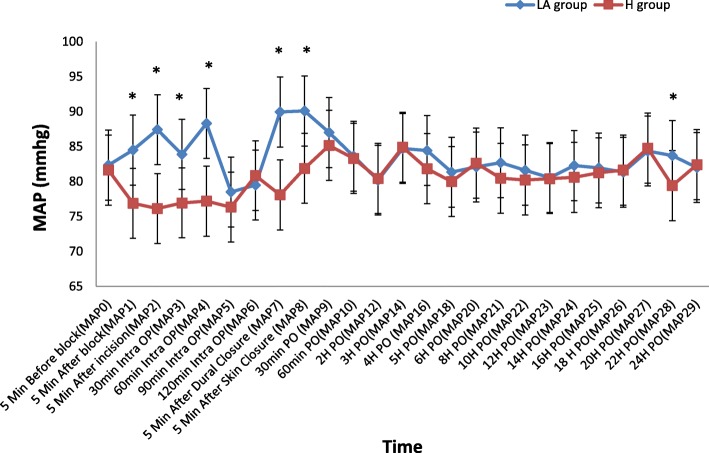
Comparison between the 2 groups as regards perioperative and intraoperative mean arterial blood pressure (MAP). *(H) Meaning for the hyaluronidase group, (LA) Meaning for the local anesthetics only group, Values are represented as mean ± SD,* * Significant P value less than (0.05), comparison between the 2 groups

- HR: There were significant statistical differences in the heart rate readings in the early intraoperative period, where they were lower in the hyaluronidase group: 5 min. After the block, 5 min after the skin incision, 30 min., 60 min. Intraoperative, 5 min. Before dural closure and 5 min. After skin closure. They continued to be significantly lower in the hyaluronidase group starting from 30 min. to 6 h postoperative. (Fig. 2)
- SBP: followed the same changes in the heart rate
- DBP: followed the same changes as HR during the intra operative period; however, there were no significant statistical differences in the entire postoperative period.
- MAP: The same as the DBP, the 22-h reading in addition was significantly lower in the hyaluronidase group, there were no statistical difference in the remaining other readings (Fig. 3)

### As regard rescue analgesia

The number of patients requiring rescue dose(s) of opioids intraoperative were significantly lower in the hyaluronidase group compared to the local anesthetics only (Table 3).

**Table 3:** Number and percent of patients required rescue analgesia

		LA group		H group		*P* Value
Rescue Analgesia	Yes	10	31.20%	2	6.2%	< 0.001*
	No	22	68.8%	30	93.8%	
First time to seek analgesia (Hours PO)		3.44 ± 1.81		6.97 ± 2.26		< 0.001*

*(H)* Meaning for the hyaluronidase group

*(LA)* Meaning for the local anesthetics only group

Values are represented as count and percent or mean ± SD as appropriate

*Significant *P* value less than (0.05), comparison between the 2 groups

### As regard the first time to ask for analgesic

The time passed for the patient to ask for analgesic was significantly lower in the hyaluronidase group compared to the local anesthetics only group (Table 3).

### As regard the effect on the patient hemoglobin level

There were no significant statistical differences between the 2 groups as regard the change in the base line hemoglobin before and after elevation of the bone flap.

### As regards interleukin-6

**Fig. 4:**
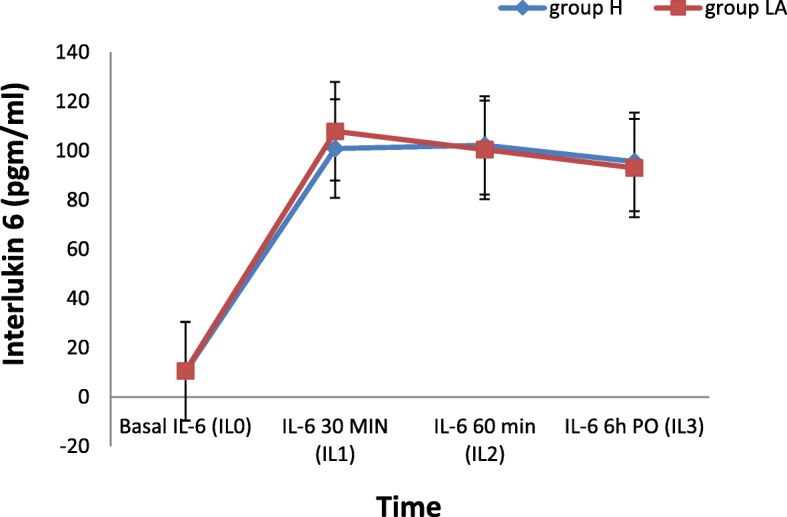
Comparison between the 2 groups as regards perioperative and intraoperative Changes in serum IL-6 levels (pgm/ml). *(H) Meaning for the hyaluronidase group, (LA) Meaning for the local anesthetics only group*

There was no statistical difference between the two groups as regard the serum levels of cortisol and interleukin-6 at the base line values, 30 min., 60 min. Intraoperative and 6 h postoperative. (Fig. 4).

### As regard the complications

- There was no serious complication reported: allergy, toxicity or profound hypotension.
- The most frequently faced complication was localized hematoma formation after injection; there were no statistically significant difference in the number of patients with this complication between the 2 groups.

## Discussion

The present study intended to evaluate the effects of adding hyaluronidase to the classic local anaesthetic mixture used in the scalp nerves block. It showed significant difference about the analgesic effects where the VAS values in the hyaluronidase group were significantly lower than the local anesthetic group in the first 8 h postoperatively, the number of patients requiring intraoperative rescue analgesic opioids was significantly lower in the hyaluronidase group as well. Moreover, the first time to seek analgesia was significantly longer in the hyaluronidase group. When looking at hemodynamics, they were favorable during the intraoperative and early postoperative periods in the hyaluronidase group especially for the HR and the SBP. On the other hand, there was no significant difference in the levels of IL-6 between both groups, and no obvious relation between IL-6 and the periods of stress nor the analgesic state. Though it was thought that hyaluronidase may increase blood loss due to its vasodilating effects [11], there was no difference in the bleeding profile as evidenced by comparable hemoglobin levels between the two groups. Furthermore, no significant observed difference in adverse effects between groups.

The results of the current study could be attributed to the action of hyaluronidase in degrading hyaluronic acid, a basic component of extracellular tissue, resulting in spreading of local anaesthetic and a better block [12]. In the modern anesthetic practice, hyaluronidase is used frequently in the ophthalmic blocks [10] recently it has been introduced to the interventions for chronic pain management [13, 14].

Several studies investigated the intraoperative and postoperative effects of adding hyaluronidase to different local anaesthetic mixtures in several blocks, however studies related to its use as adjunct to scalp nerve blocks are difficult to find. In a study by Sedghipour M. and colleagues, addition of hyaluronidase significantly improved the ocular akinesia in sub-Tenon’s anesthesia; in addition, it enhanced the intra-operative patients and surgeons’ satisfaction, and attenuated the postoperative pain as measured by VAS [10]. A further study by Chaudhari AV and colleagues investigated the effect of hyaluronidase combined to local anaesthetic in inguinal hernia block, it reported excellent and prolonged analgesia. Pain scores were statistically lower in the hyaluronidase group at 2 and 6 h postoperative as well as the number of patients requiring intraoperative analgesia [15]. Both studies are congruent with the current study as regards the favorable analgesic effects of adding hyaluronidase to local anesthetic mixtures during intraoperative and early postoperative periods.

On the other hand, Keeler JF and colleagues studied the effect of addition of hyaluronidase to bupivacaine in brachial plexus block, they reported shorter duration of the postoperative analgesia in the hyaluronidase group, and this was attributed to the enhanced absorption of the anesthetic with addition of hyaluronidase [16]. In addition, Koh WU and colleagues studied the effects of adding hyaluronidase to ropivacaine in axillary brachial plexus block and found no significant difference in the required intraoperative and postoperative analgesia between the control group and the hyaluronidase group and although the duration of postoperative analgesia was shorter in the hyaluronidase group, the result was insignificant [12]. Those results are contradictory with the current results, the different anaesthetic used, different number of patients, different dosage of drugs and different techniques of anaesthesia may explain the dissimilar outcome.

Measures of heart rate and blood pressure as a result of interaction between sympathetic and parasympathetic systems were used to assess nociception under general anesthesia, the use of local anesthetics as adjuncts to general anesthesia are supposed to improve patient response to noxious stimuli under general anesthesia [17, 18]. Tuchinda L and colleagues demonstrated a favorable effect of scalp nerves block using 2 different bupivacaine concentrations (0.5 and 0.25%) in relation to noxious stimuli as head pinning and skin incision. MAP was lower in both groups than the control group at those periods of increased stimulation [2]. Additionally, in an interesting study, Akcil EF and colleagues investigated the effects of scalp block with 0.5% bupivacaine on haemodynamics and postoperative analgesic requirements in patients undergoing infratentorial craniotomies, they compared scalp block with local anaesthetic infiltration and a control group receiving remifentanyl, they found favorable effects on haemodynamics specially at the time of pinning in the scalp block group in comparison to the other 2 groups, the scalp block as well decreased the VAS in the early postoperative period and total opioid consumption in comparison to the control group [19]. The results support the outcome of the current study as hyaluronidase may have further potentiated the effect of scalp block resulting in a better control of hemodynamics during periods of increased stress.

Conversely, a study by Torelli MJ and colleagues investigated the effects of adding hyaluronidase to mepivacaine on the heart rate and the blood pressure in patients subjected to dental surgeries. They didn’t find a statistically difference between the control group and the investigational arm. The contradiction may be due to the smaller number of drugs injected in dental surgeries, the smaller area of diffusion and the different type of local anaesthetic used [20].

M. Heesen and colleagues measured the levels of interlukin-6 during craniotomy operations, they reported rise in the plasma IL-6 during and after operations, they attributed the relatively high elevation of IL-6 due to spill out from the brain tissue after disruption of the blood brain barrier as the brain is a source of IL-6 [21]. The results of the current study are consistent with this point of view.

## Conclusions

The study may be performed in cases of awake craniotomy which may yield a more accurate assessment of the quality of the block as well as its onset which was missing in the current study. Further studies may be conducted with different doses of local anaesthetic to find out the best combination. The size of the incision and exact duration of surgery should be added in subsequent studies.

To sum up, the present data suggest that addition of hyaluronidase to the classic mixture of local anesthetics improves the quality of the block. It provides better control of the hemodynamics especially during the stressful periods such as dural incision and skin closure, thus, decreasing the need for additional doses of opioids with subsequent potential adverse effects. The analgesic effect extends into the early postoperative allowing smooth recovery and delaying the first analgesic request. No evident undesirable effects while using hyaluronidase as adjunct to local anaesthetic during scalp nerve blocks.

## Abbreviations

ASA: American society of Anesthesiology
ECG: Electrocardiogram
GABA: Gama amino butyric acid.
H: Hyaluronidase group
VAS: Visual Analogue Scale
